# Methylphenidate improves executive functions in patients with traumatic brain injuries: a feasibility trial via the idiographic approach

**DOI:** 10.1186/s12883-020-01663-x

**Published:** 2020-03-19

**Authors:** Samir Al-Adawi, Aziz Al-Naamani, Sanjay Jaju, Yahya M. Al-Farsi, Atsu S. S. Dorvlo, Ali Al-Maashani, Sara S. H. Al-Adawi, Ahmed A. Moustafa, Nasser Al-Sibani, Musthafa M. Essa, David T. Burke, M. Walid Qoronfleh

**Affiliations:** 1 Department of Behavioural Medicine, College of Medicine and Health Sciences, Sultan Qaboos University, Muscat, Oman; 2 Department of Family Medicine and Public Health, College of Medicine and Health Sciences, Sultan Qaboos University, Muscat, Oman; 3 Department of Mathematics and Statistics, College of Science, Sultan Qaboos University, Muscat, Oman; 4 Department of Neurosurgery, Khoula Hospital, Ministry of Health, Muscat, Oman; 5 Oman Medical Specialty Board, Muscat, Oman; 6 School of Social Sciences and Psychology, Marcs Institute of Brain and Behaviour, Western Sydney University, Penrith, NSW Australia; 7 Department of Food Science and Nutrition, College of Agricultural and Marine Sciences, Sultan Qaboos University, Muscat, Oman; 8 Department of Rehabilitation Medicine, Emory University School of Medicine, Atlanta, GA USA; 9 Research & Policy Department, World Innovation Summit for Health (WISH), Qatar Foundation, P.O. Box 5825, Doha, Qatar

**Keywords:** Psychopharmacology, Executive functioning, Traumatic brain injury, TBI, Cognition, Depression, Anxiety, IQCODE, Methylphenidate, Ritalin

## Abstract

**Background:**

Road traffic accidents are known to be the main cause of traumatic brain injury (TBI). TBI is also a leading cause of death and disability. This study, by means of the idiographic approach (single-case experimental designs using multiple-baseline designs), has examined whether methylphenidate (MPH - trade name Ritalin) had a differential effect on cognitive measures among patients with TBI with the sequel of acute and chronic post-concussion syndromes. The effect on gender was also explored.

**Methods:**

In comparison with healthy controls, patients with TBI (acute and chronic) and accompanying mild cognitive impairment (MCI) were screened for their integrity of executive functioning. Twenty-four patients exhibiting executive dysfunction (ED) were then instituted with the pharmacological intervention methylphenidate (MPH). The methylphenidate was administered using an uncontrolled, open label design.

**Results:**

The administration of methylphenidate impacted ED in the TBI group but had no effect on mood. Attenuation of ED was more apparent in the chronic phases of TBI. The effect on gender was not statistically significant with regard to the observed changes.

**Conclusions:**

To our knowledge, this is the first feasibility trial from the Arabian Gulf to report the performance of a TBI population with mild cognitive impairment according to the IQCODE Arabic version. This investigation confirms anecdotal observations of methylphenidate having the potential to attenuate cognitive impairment; particularly those functions that are critically involved in the integrity of executive functioning. The present feasibility trial should be followed by nomothetic studies such as those that adhere to the protocol of the randomized controlled trial. This evidence-based research is the foundation for intervention and future resource allocation by policy- or public health decision-makers.

## Background

At least 1 million people from all across the world sustain traumatic brain injury (TBI) each year [1]. Accompanying this statistic is an increasing recognition that TBI is a leading cause of death and disability [2–4] as well. Importantly, TBI typically seems to impact individuals under the age of 45, a prime age for maximum human productivity [5]. Considering this fact, the situation is likely to be even more critical to the World Health Organization (WHO) classified ‘Eastern Mediterranean’ country of Oman, where 88.6% of the country’s population falls within this age group [6].

In many Eastern Mediterranean countries, including Oman, road traffic accidents are known to be the main cause of TBI. It is estimated that road traffic accidents account for about 42 to 95% of all reported TBI cases. They are also a leading reason for death [7]. Each year, approximately 300 to 400 per 100,000 people in Oman suffer from TBI [7], translating roughly to a rate of 17 to 23 people per day. In a survey conducted by the Omani Ministry of Health [8], about 116 of the 17,791 individuals surveyed were involved in a “*serious accident*” in the preceding year. Approximately 11% of the 116 subjects were admitted to a hospital “for a long period”, and at least 1.2% currently live with varying degrees of physical handicaps, including disability. Consequently, there has been growing interest in the field of disabilities and rehabilitation amongst brain-injured populations. In addition to various physical complications, a significant number of these patients struggle with cognitive impairments, particularly impairment of executive function [9], indicated to have poor prognostic indicators compared to those with amnesiac features [10]. However, there is a dearth of studies reporting this problem conducted in the Eastern Mediterranean despite the high rate of acquired brain injuries in the region [11].

Research from other populations have revealed that executive functioning can be manipulated by agents having an affinity for dopaminergic and noradrenergic systems [12–14]. In this regard, one of the most commonly used agents are methylphenidate (MPH-Ritalin): a neuro-stimulant postulated to increase synaptic dopamine and noradrenaline levels by blocking their reuptake, accordingly, heightening information processing [15]. Emerging views suggest a neurobiological and neurochemical basis for executive functioning involving the cortico-striatal-thalamo-cortical circuits [16, 17], as well as dopaminergic and noradrenergic systems [18, 19]. In support of this explanation, preclinical animal studies have shown that disrupting catecholaminergic projections to the frontal cortex results in deficits in working memory and executive functioning [20]. Damage to the frontal cortex may be expressed as impairments in working memory, self-directed, executive, cognitive functions including the reduction in the speed of information processing and concentration. The effects of frontal impairments due to TBI are pervasive as rehabilitation and quality of life (QoL) are likely to be impacted by cognitive deficits rather than overt physical disabilities.

Studies from developed countries show that the ‘victim’ of TBI tends first to receive critical care and later undergo neurorehabilitation in dedicated units where neuro-stimulants are instituted depending on the clinical and cognitive symptoms [4]. While neuro-stimulants are widely used to tease out their impact functional recovery [12–14], with few exceptions, there is a scarcity of studies shedding light on when is the most optimal time to commence initiation of neuro-stimulants. In the literature, the time elapsed since the injury is often reported to have been shown to range from a day to several years after initial TBI injury [21, 22]. There is no description of whether the participants were treated with methylphenidate during an acute or chronic post-TBI phase. Kaelin, Cifu & Matthies [23] have operationalized acutely brain-injured adult those whose time since injury is approximately 2 months. Studies are therefore needed to shed light on whether acute (≤ 2 months) or chronic phase (≥ 2 months) is more responsive to neuro-stimulants.

In a TBI animal model, female sex hormones have been indicated to be critically involved in the modulation of dopaminergic and noradrenergic systems [24]. This would imply that there are likely to be a gender-specific response to compounds that have an affinity to dopaminergic and noradrenergic activities such as methylphenidate. In the clinical literature, investigations have suggested that there are gender-specific responses to methylphenidate [25, 26], but these studies are limited to non-TBI clinical populations. There is a lack of studies that have examined whether there are gender-specific responses to methylphenidate in the TBI population in the Eastern Mediterranean region and Oman is no exception. With the aforementioned literature review, this study has embarked to address the following aims: (i) to quantify whether treatment with methylphenidate improves executive functioning and mood in Omani patients with TBI and mild cognitive impairment and executive dysfunction, (ii) to measure the effect of methylphenidate on executive functioning and mood in acute or chronic phases of TBI, and (iii) to explore if the administration of methylphenidate and gender has a direct bearing on whether the patient is in the acute vs chronic phase. Previous studies have teased out the role of methylphenidate and cognitive enhancement. Most of the research on the TBI population are marred by a highly heterogeneous sample in the midst of heterogeneity of clinical and neuroimaging data [12, 13, 19, 21, 23]. This investigation is, therefore, constitute a feasibility trial with well defined executive dysfunction profiles that employed the idiographic approach, which is, single-subject experimental research designs using multiple-baseline designs (AABAA) that is equipped to accommodate the diversity and complexity of the TBI population [27].

## Methods

### Setting

Tertiary care is largely compartmentalized and centralized in Oman. The study sample consisted of Omani patients up to the age of 35 years who had sustained TBI and were referred to state-run tertiary care facilities at Sultan Qaboos University Hospital and Khoula Hospital for evaluation and treatment. Patients with TBI were prospectively enrolled between October 2015 and December 2016. The present study, therefore, constitutes a feasibility trial among TBI patients who were clinically evaluated as being amenable to routine pharmacological intervention.

### Participants

**Fig. 1:**
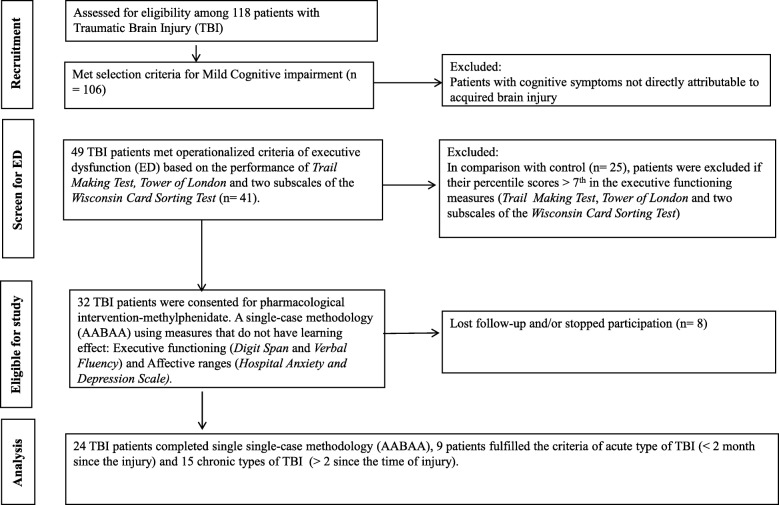
Flow diagram of study participants

For ethical approval see the such annotated section below for further consent and trial details. Also, Fig. 1 shows the study flowchart. Participants were identified clinically as being marked with cognitive impairment and presented pervasive impairment in learning and memory both in therapy and in their daily lives. The study inclusion criteria included participants with a documented history of a single TBI incident. TBI is defined here, as an injury to brain tissues caused by an external mechanical force, evidenced by a loss of consciousness, post-traumatic cognitive and behavioral changes or an objective neurological finding that can reasonably be attributed to the TBI on a physical or cognitive and behavioral status examination [28]. The second inclusion criteria are availability time since injury and other clinical risk factors as detailed in Table 1. The third inclusion criteria are participants fulfilling the criteria of mild cognitive impairment that would be selected via objective measure. The fourth inclusion criteria are that the participants manifest executive dysfunction. This was quantified using established neuropsychological measures where their percentile scores are ≤7 in the executive functioning batteries. Executive functioning is a cognitive process that involves planning, decision making, error correction and troubleshooting [29]. It also involves situations where responses are not well-rehearsed, situations that require novel sequences of actions, and that require the overcoming of a strong habitual response or resisting temptation. Another final study inclusion is complete baseline data on the “Capacity to Consent to Treatment Instrument” [30].

**Table 1:** Comparative assessment of executive functioning between traumatic brain injured Omani patients having mild cognitive impairment and healthy controls (*n* = 49)

Instrument		TBI (*n* = 24)	Control(*n* = 25)	P-value
Executive Functioning	Total score of *Trail Making Test*	156.75 ± 76.4	90.16 ± 13.29	< 0.0001
	Categories completed from *Wisconsin Card Sorting Test*	1.50 ± 0.72	3.72 ± 0.46	< 0.0001
	Perseverative errors from *Wisconsin Card Sorting Test*	17.42 ± 6.14	3.12 ± 3.44	< 0.0001
	*Tower of London*	10.00 ± 4.86	19.64 ± 5.35	< 0.0001

Analyses were conducted using student’s t-test

Exclusion criteria include those with cognitive symptoms that are not directly attributable to the acquired brain injury. Similarly, the exclusion criterion included pre-injury, psychiatric or neurological histories other than those resulting from TBI and those presently on anti-seizure medication.

### Assessment of cognitive and affective ranges

#### Cognitive functioning: mild cognitive functioning

The presence of mild cognitive impairment was solicited via a validated Arabic version of the *Informant Questionnaire on Cognitive Decline in the Elderly* (IQCODE) [31]. As per established protocol, the accompanying family, who had been living with the respondent, was asked to fill out the IQCODE [31]. The items in the IQCODE require the family member to identify what the individual was like before the TBI incident and to compare that with what the person is like now. The IQCODE is equipped to screen for the variation in cognitive functioning, which in turn suggests the presence of cognitive decline. The IQCODE-16 utilized the following scoring system: ‘major improvement’ = 1, ‘minor improvement’ = 2, ‘did not change much’ = 3, ‘minor deterioration’ = 4 and ‘major deterioration’ = 5 [32]. For the present study, only patients with TBI endorsed to have ‘minor deterioration’ were included. Existing literature shows that ‘minor deterioration’ in IQCODE denotes mild cognitive impairment. The IQCODE has been established with high accuracy to identify the presence of mild cognitive impairment similar to other well-established examinations [33, 34].

#### Cognitive functioning: executive functioning

In the neuropsychological literature, the *Wisconsin Card-Sorting Test*, *Tower of London* and *Trail Making Test* are all considered essential for assessing some of the broader domains of executive dysfunction [35]. The modified *Wisconsin Card-Sorting Test* was used to gauge “set-shifting” and cognitive flexibility via two sub-scales: “Number of Categories Completed” and “Preservative Errors”. The protocol for administering and scoring this test has been detailed in prior studies [36]. The *Tower of London Test* was employed to detect deficits in planning and the temporal organization of behavior [29, 37]; administration and scoring of this test have also been detailed in other studies [30, 38]. Lastly, the *Trail Making Test* was used to tap into cognitive processing speed, an integral part of executive functioning [39]. The scoring for the *Trail Making Test* was derived from two versions of the test, which were described elsewhere [35].

*Digit Span* (Wechsler Adult Intelligence Scale Sub-test) is a scale with two components: *Digits Forward* and *Digits Backward* [40]. In the present context, only *Digits Backward* was employed; here the participant was read a random series of numbers and asked to repeat them in the reverse order (e.g.*,* 9–1-3, should be repeated as 3–1-9). There is evidence to support the notion that *Digits Backward* rather *Digits Forward* is relevant to the integrity of executive functioning [41–43].

Verbal Fluency (The Controlled Oral Word Association Test) [44] examines the initiation and speed of verbal responses. Participants were asked to produce as many different words that begin with three specific letters as possible, with one minute allowed per letter. Subjects were explicitly told not to repeat the same word twice, and not to use words that share a common root with a previously mentioned word (for example, fish, fishing, fisherman). Following these instructions, an example and a demonstration were provided so that the participants understood the task requirements, after which he/she was given each letter in turn. The total score for Verbal Fluency is the total number of different acceptable words produced across the three 60-s periods [36]. In this experiment, the letters consisted of taa, raa, and waaw from the Arabic alphabet, as described in prior research [45]. The efficiency of verbal fluency has been linked to the integrity of the dorsolateral prefrontal cortex [46, 47].

### Affective ranges: anxiety and depressive symptoms

In order to rule out whether methylphenidate had a specific impact on executive functioning, the *Hospital Anxiety and Depression Scale* (HADS) [48] was administered to ascertain whether changes in the other measures were paralleled by alteration in anxiety and depression. The scale is a questionnaire composed of 14 items of which seven relating to depression while the other seven relating to anxiety. The psychometric properties of HADS were previously reported in the Omani population [49, 50].

### Socio-demographic and clinical risk factors

In addition to cognitive and affective ranges, various socio-demographic and clinical data were sought from the medical records or from the accompanying family member(s) or the patients themselves including gender, age, education (‘still in educational setting’, ‘completed college’, ‘some college’, ‘secondary’, ‘primary’), injury severity using *Glasgow Coma Scale* [51] (GCS; ‘mild = 13 to 15’, ‘moderate = 9 to 12’, ‘severe = less than 9’), and causes of TBI (‘road traffic accident’, ‘fall’). The presence of neuropathology if any were reported in terms of brain scans (computerized tomography-CT and magnetic resonance imaging-MRI). The time since injury was also sought, which as defined as acute (< 2 months since the TBI) or chronic (≥ 2 months since the TBI) [52–56].

### Quantification of mild cognitive functioning and executive dysfunction

In Oman and Arabic speaking population in general, there are no established measures to establish the presence of executive dysfunction. Therefore, the protracted exercises were needed to establish the presence of executive dysfunction. For quantification of mild cognitive impairment, the *Informant Questionnaire on Cognitive Decline in the Elderly* (IQCODE), was used. As shown in Fig. 1, IQCODE identified 106 out of 118 to meet the selection criteria for mild cognitive impairment.

The recognized 106 patients they were further scrutinized for the presence of executive functioning via their performance on the *Wisconsin Card-Sorting Test*, *Tower of London Test* and *Trail Making Tes*t that are known to be the *sine-qua non* for assessing executive functioning. Healthy volunteers were therefore recruited in order to establish the extent to which the TBI subjects with mild cognitive impairment ‘deviated’ from the healthy control group. The control population was recruited from amongst the staff of Sultan Qaboos University-SQU (*n* = 25). The healthy controls were matched for age, gender, and education. Inclusion for the control group included those with no history of medical, psychiatric or neurological conditions that warranted medical attention. This was orally confirmed for all consenting participants.

In order to quantify whether the TBI group exhibited executive dysfunction, z-values were computed for both the TBI and healthy control cohorts. Domain scores for each subject were calculated based on the mean test results of all tests from the respective domain. As detailed in other studies [29], tests were defined as indicating cases of executive dysfunction if the z-value was less than − 1.5. A domain was judged as pathological if more than half of the included tests of the respective domain showed z-values below − 1.5 or percentile <7th. Table 2 highlights significant differences between the TBI group and the control group on the four administered tests for assessment of Executive Functioning (*Trail Making Test*, *Tower of London Test* and two sub-scales of the *Wisconsin Card Sorting Test*). In the *Trail Making Test*, the control group had an average of 90 s (SD = 13), while the TBI group took substantially longer (average of 157 s (SD = 76) to complete the task (*p* < 0.001). In the *Wisconsin Card Sorting Test*, “Number of Categories Completed” and “Preservative Errors” were determined. In the former, TBI patients completed only 1.5 categories on average, which is significantly less than the control group who completed an average of 3.7 categories (p < 0.001). The patient group also had significantly more “Preservative Errors” compared to the control (17 vs 3; p < 0.001). In the *Tower of London Test*, the control group scored nearly twice as high as the TBI patients (19.6 vs 10; p < 0.001) (Table 1). Therefore, as shown in Fig. 1, the participants with TBI (*n* = 49) met operationalized criteria of executive dysfunction.

**Table 2:** Clinical and demographic characteristics of traumatic brain injured Omani participants with mild cognitive impairment and executive dysfunction

Parameter		No. of Patients	% of Patients
Gender	Male	13	54.2
	Female	11	45.8
Age	18–25	17	70.8
	26–35	7	29.2
Education	Still in Educational setting	5	20.8
	Completed College	3	12.5
	Some College	2	8.3
	Secondary	11	45.8
	Primary	3	12.5
Injury severity	Unknown	3	12.5
	GCS (mild; 13 to 15)	5	20.8
	GCS (moderate; 9 to 12)	15	62.5
	GCS (severe; less than 9)	1	4.2
Causes of Traumatic Brain Injury	Unknown	2	8.3
	Road Traffic Accident	20	83.3
	Fall	2	8.3
Time since injury	Acute (< 2 months)	9	37.5
	Chronic (≥ 2 months)	15	62.5
Neuropathology (CT or MRI)	Diffused	6	25
	Bilateral Frontal Hematoma	12	50
	Focal	6	25

*CT* = Computed Tomography Imaging; *MRI* = magnetic resonance imaging; *GSC* = Glasgow Coma Scale

### Protocol for a feasibility trial

Participants with TBI (*n* = 32) with z-values below − 1.5 were deemed to suit the study criteria for feasibility trial with methylphenidate (MPH) using AABAA single-case methodology as depicted below graphically (Table 3). The methylphenidate was administered using an uncontrolled, open label design which is explained below.

**Table 3:** Protocol design for the administration of methylphenidate that followed AABAA single case methodology

A	Baseline 1 (BL1)
^*^A	Baseline 2 (BL2)
^**^B	Maximum dose of methylphenidate (MAXMETH)
^***^A	Post-withdrawal 1 (POST1)
^***^A	Post-withdrawal 2 (POST2)

* BL2 evaluations conducted over 15 days of BL1 and then the institution of drug treatment (the starting dose was 5 mg/day, increasing to a maximum dosage of 10 mg/day)

** MAXMETH evaluations conducted at 30 days after BL2

*** Follow-up evaluations (POST1) were conducted at 15 days after the MAXMETH evaluation while POST2 was conducted after 15 days after POST1

The assessments were conducted at baseline after the maximum dose of methylphenidate was instituted and the withdrawal of methylphenidate. Both *Digit Span* and *Verbal Fluency* circumvent the issue of practice or learning effect owing to the availability of various versions of these two measures and hence were used for successive assessments of the TBI patients. The baseline (BL) performance on cognitive measures constituted the average of two consecutive scores of the *Digit Span* and *Verbal Fluency* and similarly, the average of post-methylphenidate withdrawal (POST) performances was considered. The cognitive performance scores at a maximum dosage of methylphenidate were considered only once. Hospital Anxiety and Depression (HADS) was utilized to solicit the variations in anxiety and depression symptom in parallel with cognitive measures.

For the purpose of analysis of their responses, the patients were divided into two groups based on the time elapsed since the injury. Those whose time since injury was less than two months were operationalized as ‘acute’ TBI and the rest were operationalized as ‘chronic TBI’ [52]. The basic single-case methodology (AABAA) adopted was a repeated measure, with multiple baselines as shown above schematically. The second baseline assessments (A) was one week after the first baseline (A) and then (B) instituting methylphenidate (the starting dose was 5 mg/day, increasing to a maximum dosage of 10 mg/day). The assessment (B) took place when participants were in methylphenidate, that is, 30 days after the second baseline. Follow-up evaluations (POST1) were conducted at 15 days after the MAXMETH evaluation while POST2 was conducted after 15 days after POST1.

### Statistical analysis

Statistical analysis was performed using SPSS Version 19. Summary tables were produced. *t*-tests and repeated *ANOVA* tests were used and *p*-values were reported in the tables.

### Ethical approval, trial withdrawal and discontinuation of trial medication

This study was approved by the Institutional Review Board, ‘Ethical Committee for Human and Clinical Research’ and the ‘Medical Research Committee’ (Project No. MED 99–4) of of the College of Medicine & Health Sciences, Sultan Qaboos University (SQU). Participants were requested to provide written informed consent which was also confirmed verbally again prior to data collection, and the study procedures were carried out in accordance with the Code of Ethics of the World Medical Association (Declaration of Helsinki of 1964–2008) for human experiments including confidentiality, privacy, documentation and data management. As for the trial withdrawal and discontinuation of trial medication, participants were explicitly informed that they are free to withdraw from the trial at any time without reason or impact on usual care. As shown in Fig. 1, only data from completed cycles were included in the analysis.

## Results

Table 2 details clinical and demographic information of the TBI patients identified as having executive dysfunction (Fig. 1 provides the flowchart). Of the 32 patients, 24 completed the study (11 were female and 13 were male), however, 8 were lost at follow-up and/or stopped participation. Out of the 8, 4 could not tolerate the medication. The majority had received education up to secondary school and above (*n* = 21). In terms of severity of the injury, 15 patients had moderate scores and 5 had mild scores on the *Glasgow Coma Scale* [57]. Neuro-imaging data indicated that 25% (*n* = 6) of the TBI group exhibited characteristics of diffused and focal brain injuries respectively, while 50% (*n* = 12) had frontal hematoma. Road traffic accidents were the major cause of TBI in this group (20 out of 24). Almost 63% of the cases were chronic cases. Nearly 50% had bilateral frontal hematoma as evidenced by computed tomography imaging or magnetic resonance imaging.

Further assessments of all TBI patients using stable cognitive measures (*Verbal Fluency*, *Digit Span*) and affective measures (anxiety and depression) across different time periods in comparison to baseline values are shown in Table 4. There was a significant rise in *Digit Span* results. A baseline reading of 4.00 (SD 0.81) increased to 5.71 (SD 0.81) following the administration of methylphenidate. The scores remained high at 5.88 (SD 1.87), post-methylphenidate withdrawal (*p* < 0.001 in both situations). *Verbal Fluency* similarly exhibited a significant increase from the baseline score (9.33, SD 2.98) to scores obtained during methylphenidate treatment (12.38, SD 3.41; p < 0.001) and post-withdrawal scores (12.21, SD 4.31; *p* = 0.008).

**Table 4:** Measures of executive functioning (*Verbal Fluency, Digit Span*) and affective measures (HADS-*Hospital and Depression Scale*) across assessment occasions (baseline, during methylphenidate and post-withdrawal of methylphenidate) among a sample of Omanis with traumatic brain injury

Category	Measure	Assessment	Min	Mean	Max	Std. Dev	p
Executive Functioning	Digit Span	Average baseline	3	4.00	6	0.81	
		with methylphenidate	4	5.71	7	0.81	p < 0.001
		Average Post withdrawal	3	5.88	8	1.87	p < 0.001
	Verbal Fluency	Average baseline	3	9.33	16	2.96	
		with methylphenidate	7	12.38	18	3.41	p < 0.001
		Average Post withdrawal	5	12.21	19	4.31	P = 0.008
Affective measures	HADS-Depression	Average baseline	2	7.02	13	2.63	
		with methylphenidate	2	6.50	13	2.69	p = 0. 033
		Average Post withdrawal	2	6.54	13	2.89	p = 0. 150
	HADS-Anxiety	Average Baseline	2	6.77	15	3.45	
		with methylphenidate	2	6.88	15	3.31	*p* = 0.732
		Average Post withdrawal	2	6.67	15	3.31	*p* = 0.719

*All comparisons indicated were with the baseline assessments

The average HADS–Depression score of 7.02 (SD 2.63) at baseline decreased to 6.50 (SD 2.69) with methylphenidate administration (*p* = 0.033) but showed no significant change post-methylphenidate withdrawal with a score of 6.54 (SD 2.89) (*p* = 0.150). There was no significant change in the baseline result of anxiety (6.77, SD 3.45) from the results obtained after methylphenidate administration (6.88) and post-withdrawal (6.67).

Table 5 and Table 6 describe raw scores to examine whether acute (< 2 months) or chronic phases of TBI (≥ 2 months) had any effect on executive functioning and mood after administration and later withdrawal of methylphenidate. Nine patients were in the acute phase of TBI during the study, these were seen less than two months after injury (four males and five females), and 15 were in the chronic phase of TBI, they were seen two or more months after the injury (nine males and six females). Based on their scores, both groups showed improvement in executive functioning following methylphenidate intervention. When executive functioning was assessed post-withdrawal, it was found that the acute group reverted to their original baseline scores, while the chronic group maintained the improved scores. Detailed analysis revealed that the TBI phase (i.e. *acute* vs *chronic)* had a substantial effect on executive functioning (*Digit Span* and *Verbal Fluency)*, especially after correcting for baseline values (Table 7). *Digit Span* and *Verbal Fluency* scores were significantly higher than post-withdrawal scores for the chronic group, implying that methylphenidate enhanced executive functioning. The changes in HADS–Depression and HADS–Anxiety across the two TBI phases were not significant (Table 7).

**Table 5:** Descriptive statistics of executive functioning (*Digit Span*, *Verbal Fluency*) among a sample of Omanis with traumatic brain injury and accompanying mild cognitive impairment between time since injury and across different assessment periods based on gender

Executive functioning		Time since injury	Baseline^a^ Mean, SD	Methylphenidate^b^ Mean, SD	Post withdrawal^c^ Mean, SD
Digit Span	Total	< 2 months	3.67 ± 1.00	5.22 ± 0.83	3.67 ± 0.71
		≥ 2 months	4.20 ± 0.86	6.00 ± 0.65	7.20 ± 0.68
	Females	< 2 months	3.60 ± 1.34	5.40 ± 0.89	3.80 ± 0.84
		≥ 2 months	4.00 ± 1.09	6.00 ± 0.89	7.17 ± 0.75
	Males	< 2 months	3.75 ± 0.05	5.00 ± 0.82	3.50 ± 0.58
		≥ 2 months	4.33 ± 0.70	6.00 ± 0.50	7.22 ± 0.68
Verbal Fluency	Total	< 2 months	10.00 ± 2.60	12.44 ± 2.83	8.00 ± 1.66
		≥ 2 months	9.07 ± 3.30	12.33 ± 3.81	14.73 ± 3.28
	Females	< 2 months	8.80 ± 2.05	11.60 ± 2.97	7.80 ± 1.09
		≥ 2 months	8.33 ± 3.61	11.00 ± 3.41	13.50 ± 4.18
	Males	< 2 months	11.50 ± 2.65	13.50 ± 2.65	8.25 ± 2.36
		≥ 2 months	9.56 ± 3.20	13.22 ± 3.99	15.56 ± 2.45

^a^ Averages score across two baseline assessments

^b^ Average scores across two post-intervention assessments

^c^ The starting dose was 5 mg/day, increasing to a maximum dosage of 10 mg/day

**Table 6:** Descriptive statistics of affective functioning (HADS-*Hospital Anxiety and Depression Scale*) among a sample of Omanis with traumatic brain injury suffering from mild cognitive impairment between time since injury and across different assessment periods based on gender

Affective functioning		Time since injury	Baseline Mean, SD	Methylphenidate Mean, SD	Post withdrawal Mean, SD
HADS - Depression	Total	< 2 months	7.90 ± 3.73	7.33 ± 3.35	6.78 ± 4.05
		≥ 2 months	6.50 ± 1.61	6.00 ± 2.17	6.4 ± 2.06
	Females	< 2 months	5.20 ± 2.59	5.20 ± 2.49	4.40 ± 2.88
		≥ 2 months	6.0 ± 1.41	5.67 ± 2.06	5.83 ± 1.32
	Males	< 2 months	11.25 ± 1.26	10.00 ± 2.16	9.75 ± 3.40
		≥ 2 months	6.67 ± 1.73	6.22 ± 2.33	6.78 ± 2.44
HADS - Anxiety	Total	< 2 months	5.44 ± 3.64	6.00 ± 3.35	5.89 ± 3.48
		≥ 2 months	7.40 ± 3.31	7.40 ± 3.29	7.13 ± 3.22
	Females	< 2 months	5.40 ± 3.78	6.40 ± 3.13	6.20 ± 3.42
		≥ 2 months	9.00 ± 3.95	8.50 ± 3.62	8.50 ± 3.62
	Males	< 2 months	5.50 ± 4.04	5.50 ± 4.04	5.50 ± 4.04
		≥ 2 months	6.33 ± 2.50	6.67 ± 3.04	6.22 ± 2.77

**Table 7:** Effect of Methylphenidate on Executive (*Digit Span and Verbal Fluency*) and Anxiety and Depression tapped by Hospital Anxiety and Depression Scale (HADS)

Classification	During the treatment period	Post withdrawal period
	*P*-value^*^using the baseline as covariate	
Digit Span	0.051	≤0.001
Verbal Fluency	0.334	≤0.001
HADS - Depression	0.933	0.169
HADS - Anxiety	0.625	0.415

*P*-values for comparisons over the time since injuryand Depression Scale (HADS) across gender

Gender did not contribute to any significant difference in the scores of executive functioning and mood during methylphenidate treatment and after its withdrawal (Table 8).

**Table 8:** Effect of Methylphenidate on Digit Span and Verbal Fluency (Executive) and Anxiety and Depression from Hospital Anxiety and Depression Scale (HADS) across gender.

Classification	During the treatment period	Post withdrawal period
	P-value* **using the baseline as covariate**	
Digit Span	0.677	0.895
Verbal Fluency	0.400	0.237
HADS - Depression	0.492	0.614
HADS - Anxiety	0.891	0.824

*P-values for comparing males against females

## Discussion

This feasibility trial has specifically employed the idiographic approach due to the nature of the TBI population whereby accruing a large sample size with the stable and homogeneous clinical data for neuropsychological evaluation and the pharmacological challenge is often untenable [58]. Such a cohort is, therefore, more apt to be studied using idiographic than a nomothetic approach. For example, as for the present cohort, 20% of the patients were mild TBI and 62.5% were classified as moderate. In addition, only one patient experienced a severe TBI status. It is worth mentioning, that each injury level has a distinctly different prognosis and presentation by CT/MRI. Moreover, 37% of the patients were in the acute phase while 63% were in the chronic phase of injury. This feasibility trial explored three interrelated themes using idiographic–AABAA single experimental design.

The first aim was to investigate whether a sample of TBI patients would perform differently on measures of executive functioning when compared to a healthy population in Oman. Attributes that are most often viewed as defining a human being (e.g., reasoning, temporal organization of behavior, self-regulation, mental flexibility, aspects of attention and awareness) are some of the primary constituents of executive functioning. Furthermore, there is extensive empirical literature suggesting that executive dysfunction is one of the most debilitating and intransigent aspects of cognitive impairment typically acquired during traumatic brain injury [59, 60]. To our knowledge, this is the first feasibility trial from the Arabian Gulf to report the performance of a TBI population with mild cognitive impairment performing poorly on measures of executive functioning compared to a healthy control group, according to an Arabic version of *Informant Questionnaire on Cognitive Decline in the Elderly* (IQCODE) [32]. In prior studies, there has been a debate on whether mild cognitive impairment is a prodromal stage of neurogenerative disorders or ‘normal’ variation of cognitive functioning [61]. In the general population, the constituent of mild cognitive impairment has been postulated to include ill-defined memory impairment, intact global cognitive and intellectual capacity and no overt symptoms of dementia [62]. This feasibility trial suggests that in a TBI population, there is impairment of executive functioning among those who fulfilled the criteria of mild cognitive impairment, a view previously noted by others [63, 64].

Huang et al. [58], in their meta-analysis, have identified 683 published studies that have reported the use of methylphenidate for the TBI population from electronic databases. These studies employed the spectrum of cognitive measures. The authors concluded that methylphenidate has the potential to mostly enhancing attentional capacity but not memory or processing speed. There have been indications that some pharmacological approaches may be useful in addressing aspects of executive dysfunction [65, 66], but no studies have been forthcoming from the Eastern Mediterranean. This feasibility trial suggests that treatment with the catecholaminergic agonist, methylphenidate, impacted executive dysfunction. Averaging across all participants, methylphenidate treatment did not produce any significant changes in mood, suggesting that pharmacological intervention impacted cognitive functioning directly rather than via the patients’ affected state. Discussions in the current literature on whether cognitive functioning is influenced by mood or, conversely, whether the mood is influenced by the level of cognitive functioning, are ongoing [67, 68]. This interpretation is consistent with systematic and meta-analytic reviews suggesting the lack of effectiveness of methylphenidate in mitigating symptoms of depression [69]. It is worthwhile to note that mood state was measured using the ‘symptom checklist’, HADS. Future studies should employ more robust measures such as a semi-structured diagnostic interview, the ‘gold standard’.

The TBI population in Oman has had no access to dedicated restorative therapies [70]. An enduring theme in prior studies is the tendency for a majority of brain-injured patients to recover spontaneously. Another issue is the establishment of a window of recovery for cognitive impairment. Therefore, deciphering when to intervene is imperative. This feasibility trial indicates that time since the injury has an effect on cognitive measures (*Digit Span* and *Verbal Fluency*). These measures appear to have been robustly affected in the “chronic” cohort. In contrast, the performance on affective measures has little bearing on whether the participants belong to the “acute” or “chronic” group. Furthermore, the observed improvement with methylphenidate administration was maintained after withdrawal in only the “chronic” group, but not the “acute” group. This essentially seems to suggest that the administration of methylphenidate is more effective for the “chronic” group. This raises the question as to whether the improvement in cognitive function scores can be attributed to the effect of methylphenidate, or whether it is simply part of spontaneous recovery. Despite such a caveat, the consistent baseline scores and sudden improvement found only after pharmacological intervention suggest that the improvements were indeed attributed to chronicity and methylphenidate. Future studies might explore when it is optimal to use methylphenidate on a TBI population.

In prior work, there has been a dearth of studies examining the trajectory of time since injury, intervention, and outcome in TBI. What constitutes acute or chronic is sometimes inferred in terms of ‘primary’ and ‘secondary injury’ [53]. Primary injury has been operationalized as a time period immediately following injury [54]. Secondary damage is characterized by complications starting within minutes, hours, days or even months after primary injury and includes different events such as changes in gene expressions, inflammation, ischaemia, central immunomodulation disturbance, impaired energy metabolism and neuronal death [55, 56]. These variations of such a cascade of pathological processes have a direct bearing on the functional outcome [71]. There is some evidence to suggest that secondary injury may not be triggered directly by trauma, but rather a combination of primary injury and the resultant array of pathological processes. A clear-cut demarcation between primary and secondary injury has yet to be established. Undoubtedly, tudies to define what constitutes acute and chronic phases of TBI are needed. As this feasibility trial suggests, such demarcation might lead to prognostic indicators of when neuro-stimulants are used.

Previously, females have shown have poorer prognostic indicators and recovery compared to males [72]. This feasibility trial ostensibly discounts the issue of gender in executive functioning. The findings suggest no significant difference between male and female patients on all measures. Furthermore, it appears that methylphenidate has the potential to attenuate symptoms of executive dysfunction in both genders. Some studies shedding light on how and why methylphenidate works on executive functioning have emerged. Methylphenidate has been suggested to trigger modulation of the catecholaminergic system or specifically, its capability to attenuate reuptake of dopamine and norepinephrine neurotransmission in the brain [19]. Interestingly catecholaminergic neuron has been shown to innervate cortical and subcortical regions thought to be critically involved in executive functioning in primates [73]. In human beings, compounds with affinity to the catecholaminergic system such as methylphenidate have been shown to heighten executive functioning and its neural substrate in the human brain [64]. Thus, reflecting on prior scholarly work, the present finding largely supports the previous contention that executive functioning can be manipulated with compounds that affect dopaminergic and noradrenergic systems [74–76].

An important finding in the present feasibility trial was that the improvement did not reverse following drug withdrawal. Although this feasibility trial was not equipped to investigate the issue of pharmacologically induced neuroplasticity, there are some anecdotal and clinical observations indicating that neuro-stimulants, such as methylphenidate, have had the capacity to “kick-start” spontaneous recovery [36]. One opinion suggests brain injury triggers the cascade of excitotoxity [77, 78]. Interestingly, there is evidence to propose that such excitotoxity can be ‘scavenged’ by the catecholaminergic system [79, 80], for example, when methylphenidate is exogenously induced [77, 81].

### Limitations

Despite that non-parametric statistics were utilized to observe changes across baseline, intervention and post-withdrawal periods, some limitations of the feasibility trial ought to be highlighted. *First* and foremost, this feasibility trial is limited by being an open-label study (i.e., not double blinded and with no control group), a cohort with homogeneity of injuries and a relatively small sample size. Despite fulfilling the requirement of the idiographic study, when a feasibility trial is scrutinized under the prism of the nomothetic microscope, are likely to be seen with several limitations. Therefore, this feasibility trial should lay the groundwork for randomized clinical trials. *Secondly*, this feasibility trial could have employed another catecholaminergic agonist compound other than methylphenidate to validate findings. *Thirdly*, the generalizability of this feasibility trial might be lacking because it did not include motor and functional metrics to complement present neurocognitive metrics. Related to assessment, this feasibility trial has embarked to quantify the presence of mild cognitive impairment using IQCODE. In addition to controversies on what constitutes mild cognitive impairment [82], IQCODE lacks the vigor of the other bedside measures such as Montreal Cognitive Assessment (MoCA) and Mini-Mental State Examination (MMSE) for soliciting the presence of cognitive decline but there are dissenting views [31, 83]. IQCODE has been validated in the Arabic speaking population and appears to have good psychometric properties [31, 33, 34]. Future studies should employed other conventional neuropsychological measures to tap into the presence of mild cognitive impairment. Other than IQCODE, the present feasibility trial has utilized strong non-verbal components or those that have been employed in the Arabic-speaking population previously. With a few exceptions, the majority of the measures do not have cutoffs for the TBI population in Oman. For these reasons, protracted exercises were taken to establish ‘caseness’ for executive dysfunction. For these reasons, ‘caseness’ for executive dysfunction was derived from the z-values (− 1.5) or percentile = <7th) as previously reported elsewhere [29]. *Fourthly*, it might be theoretically interesting to include another cohort of TBI rather than those with executive functioning. This might increase the scientific merit of the current feasibility trial. *Fifthly*, in Oman, the demography is characterized by a pyramidal-like population structure with the majority as a youth. Indeed, the previous survey in Oman has indicated that the majority of the victim of the road traffic accident and TBI are youngster [84]. In a population of younger TBI patients as the present cohort, it likely that noted ‘improvements’ could stem from many factors including placebo effect, spontaneous recovery, and/ or developmental changes as the brain is well established to continue growth until ~ age 25. The present study, open-label, was not equipped to circumvent these confounders. Additional studies using a vigorous research methodology are needed to shed further light on the trajectory between time since injury, neuropsychological recovery, and the impact of a number of commonly used pharmacological agents on brain-injured populations.

## Conclusions

Despite traumatic brain injury being a global challenge, issues pertinent to the disability it triggers and the rehabilitation that is needed in emerging economies, such as Oman, have received scant attention in past literature. The present study utilized a feasibility trial to explore whether treatment with methylphenidate, in patients with TBI, would improve executive functioning. The investigation was uncontrolled, open label design. The study results suggested that methylphenidate tended to positively impact executive functioning. The study outcome also suggested that noted improvement was most heavily dictated by time elapsed since the injury. Unlike prior research, there was no gender difference in executive functioning or response to treatment. An unpredicted finding in the present study was that improvements did not reverse following drug withdrawal among those patients operationalized in the “chronic” group. Being a feasibility trial, it is important for future studies to scrutinize these present findings through a study design with a more robust methodology. Finally, we believe this is an important feasibility study in this population. In the future, we want to extend this work into randomized controlled trial (RCT) to address limitations, strengthen findings that supports the conclusion and confirmation of methylphenidate treatment. This evidence-based research is valuable to both public health officials and policy decision-makers in order to design and implement policies that improve health and social outcomes.

## Data Availability

This is a research article and all data generated or analyzed during this study are included in this published article.

## Abbreviations

CT: Computerized Tomography
ED: Executive Dysfunction
GCS: Glasgow Coma Scale
HADS: Hospital Anxiety and Depression Scale
IQCODE: Informant Questionnaire on Cognitive Decline in the Elderly
MCI: Mild Cognitive Impairment
MPH: Methylphenidate
MRI: Magnetic Resonance Imaging
QoL: Quality of Life
RCT: Randomized Controlled Trial
SD: Standard Deviation
TBI: Traumatic Brain Injury
WHO: World Health Organization
